# Lay health worker experiences administering a multi-level combination intervention to improve PMTCT retention

**DOI:** 10.1186/s12913-017-2825-8

**Published:** 2018-01-10

**Authors:** Abby DiCarlo, Ruby Fayorsey, Masila Syengo, Duncan Chege, Martin Sirengo, William Reidy, Juliana Otieno, Jackton Omoto, Mark P. Hawken, Elaine J. Abrams

**Affiliations:** 1 ICAP at Columbia University, Mailman School of Public Health, 722 W. 168th Street, New York, NY 10032 USA; 2 ICAP Kenya, Mailman School of Public Health, Columbia University, New York, NY USA; 3 National AIDS and STI Control Program, Nairobi, Kenya; 4 Jaramogi Oginga Odinga Teaching and Referral Hospital, Kisumu, Kenya; 5 School of Medicine, Maseno University, Kisumu, Kenya

**Keywords:** Prevention of mother-to-child transmission (PMTCT), Combination intervention, Kenya, Lay health workers, Retention

## Abstract

**Background:**

The recent scale-up of prevention of mother-to-child transmission of HIV (PMTCT) services has rapidly accelerated antiretroviral therapy (ART) uptake among pregnant and postpartum women in sub-Saharan Africa. The Mother and Infant Retention for Health (MIR4Health) study evaluates the impact of a combination intervention administered by trained lay health workers to decrease attrition among HIV-positive women initiating PMTCT services and their infants through 6 months postpartum.

**Methods:**

This was a qualitative study nested within the MIR4Health trial. MIR4Health was conducted at 10 health facilities in Nyanza, Kenya from September 2013 to September 2015. The trial intervention addressed behavioral, social, and structural barriers to PMTCT retention and included: appointment reminders via text and phone calls, follow-up and tracking for missed clinic visits, PMTCT health education at home visits and during clinic visits, and retention and adherence support and counseling. All interventions were administered by lay health workers. We describe results of a nested small qualitative inquiry which conducted two focus groups to assess the experiences and perceptions of lay health workers administering the interventions. Discussions were recorded and simultaneously transcribed and translated into English. Data were analyzed using framework analysis approach.

**Results:**

Study findings show lay health workers played a critical role supporting mothers in PMTCT services across a range of behavioral, social, and structural domains, including improved communication and contact, health education, peer support, and patient advocacy and assistance. Findings also identified barriers to the uptake and implementation of the interventions, such as concerns about privacy and stigma, and the limitations of the healthcare system including healthcare worker attitudes. Overall, study findings indicate that lay health workers found the interventions to be feasible, acceptable, and well received by clients.

**Conclusions:**

Lay health workers played a fundamental role in supporting mothers engaged in PMTCT services and provided valuable feedback on the implementation of PMTCT interventions. Future interventions must include strategies to ensure client privacy, decrease stigma within communities, and address the practical limitations of health systems. This study adds important insight to the growing body of research on lay health worker experiences in HIV and PMTCT care.

**Trial registration:**

Clinicaltrials.gov NCT01962220.

## Background

In 2013, the World Health Organization recommended Option B+ for the prevention of mother-to-child HIV transmission (PMTCT), an approach in which all HIV-positive pregnant and breastfeeding women initiate lifelong antiretroviral therapy (ART), independent of CD4 count. The majority of low and middle-income countries (LMIC) have endorsed Option B+, resulting in a rapid acceleration of ART uptake and coverage across sub-Saharan Africa and global plan priority countries [1]. Evidence suggests that mother-to-child HIV transmission rates are falling, with a 60% reduction in new pediatric infections since 2009 in priority countries [1].

While major progress has been made in scale-up of universal treatment for all pregnant and postpartum women, other important issues have arisen, particularly regarding the challenges of retention in PMTCT services. For example, in Malawi where Option B+ was first introduced, women who started ART during pregnancy were five times more likely to be lost to follow-up than non-pregnant ART-eligible women who initiated treatment [2]. Similar findings have been reported from many settings implementing universal treatment [3–7]. Multiple factors have been attributed to low retention rates including behavioral, social and structural factors, and various interventions have been trialed and implemented in efforts to improve retention in care among women and infants in PMTCT services [8–12].

Lifelong retention in care is a vital component of PMTCT and HIV care cascade, and the elimination of mother-to-child transmission of HIV, and improving survival of mothers and infants will require identification of effective, feasible, cost-effective, and scalable strategies to engage and retain HIV-positive pregnant women and their infants in PMTCT and HIV care and treatment programs across the PMTCT/ HIV care cascade. Previous interventions that have demonstrated improved retention in PMTCT care often implement a single intervention [13], and improved retention has been associated with interventions such as peer mentoring and counseling, mobile phone calls and text messages, home visits, and the integration of PMTCT into routine care [5, 13–23]. There is increasing recognition that combination interventions, which address multiple factors across the behavioral, social, and structural levels, may be more effective than single interventions, particularly among high-risk populations and regions where it is most important to address both the immediate risk and its underlying context [24, 25].

Healthcare workers are an essential part of the scale up and retention in PMTCT services. The shortage of healthcare workers in sub-Saharan Africa has been well established; sub-Saharan Africa is home to two-thirds of people living with HIV/AIDS globally, but only 3% of the world health workforce [26]. This shortage is estimated to grow significantly by 2030, despite current efforts to increase healthcare worker production and employment [27]. The World Health Organization has endorsed a task-shifting approach, which reorganizes and decentralizes specific health services from highly qualified and trained health professionals to health workers with fewer qualifications and less training [28]. This approach has been adopted in many resource-limited countries with high HIV burdens, leading to increasing numbers of lay health workers to expand service provision, facilitate the initiation of ART and support retention in care [29, 30]. Previous research has demonstrated that lay health workers (a health worker who performs functions related to health care delivery and is trained in some way in the context of an intervention, but who has not received a formal professional or paraprofessional certificate or tertiary education degree) play a crucial role in the delivery and retention of PMTCT services, supporting patients along all stages of the PMTCT cascade [27]. Lay health workers are specifically positioned to recognize behavioral, social, and structural issues related to engagement and retention in PMTCT that may not be obvious during scheduled clinic visits [31, 32]. While several studies have explored the use of lay health workers in HIV services in sub-Saharan Africa, more research is needed to fully understand the experiences and contributions of lay health workers, specifically in the context of PMTCT services [32].

Kenya has rapidly scaled up PMTCT services in recent years, and formally implemented Option B+ in 2014. This has led to great strides in PMTCT service coverage, with nearly 75% of HIV-positive pregnant women receiving ART in 2015 [33]. Lay health workers are an integral part of health services in Kenya. The Kenya Community Health Strategy formalized the utilization of lay health workers to promote healthy behavior and expand access to healthcare [34, 35]. In the context of the PMTCT scale-up, lay health workers are on the frontlines of PMTCT care, working directly with HIV-positive women across multiple platforms, including patient support and communication, counseling and education, monitoring and referrals.

The Mother and Infant Retention for Health (MIR4Health) study evaluated the impact of a combination intervention administered by trained lay health workers to improve retention among HIV-positive women initiating PMTCT services and their infants through 6 months postpartum [36]. The parent study was conducted at 10 health facilities in western Kenya from September 2013 to September 2015. Two focus group discussions (FGD) were conducted with the lay health workers, 1 month after all study follow-up had been completed in order to understand their experiences with and perceptions of the combination intervention.

## Methods

This study was a small qualitative inquiry nested within MIR4Health, an individualized randomized trial evaluating the effectiveness of a combination intervention to improve retention among HIV-positive women initiating PMTCT services at 10 health facilities in the Nyanza region of Kenya that offered integrated PMTCT and HIV care and treatment. The main trial enrolled 340 HIV-positive pregnant women, of which 170 women were randomized to the intervention arm and 170 received routine standard of care support services available at each facility. All trial participants received standard of care services including routine ANC, delivery, and postpartum care along with PMTCT and HIV care at the mother and child health (MCH) clinic, group health education and option to enroll in monthly psychosocial support group per Kenyan national guidelines [37]. The FGD explored appropriateness of the interventions, feasibility and potential barriers and challenges to scale-up.

### Study intervention procedures

Trial participants randomized to the intervention arm received the multicomponent combination intervention to address behavioral, social, and structural barriers to PMTCT retention. The intervention was delivered by 15 lay health workers, called *Mama Mshauri,* who were recruited from the community, hired, and trained for this study. The 15 *Mama Mshauri* cumulatively delivered the intervention to 170 HIV-positive pregnant women as part of the larger study. Each *Mama Mshauri* had an average of 22 clients. All *Mama Mshauri* underwent a week long practical training on intervention protocols, including communication skills, PMTCT care, flip-chart use, and adherence and psychosocial support. They were introduced to participants during the randomization visit, met with them at each clinic visit during the antenatal period and post-partum period, and visited assigned participants’ homes on a monthly basis until 6 months post-partum. During clinic and home visits the *Mama Mshauri* provided individualized PMTCT health education using a study specific flip chart and retention and adherence support. During the clinic visits, they assisted with expediting service provision, enhancing communication between participants and health providers, assisting participants to identify and problem-solve personal barriers to retention and adherence, and providing psychosocial support and counseling. The *Mama Mshauri *sent phone reminders 3 days before the clinic visit and SMS reminders 7 days and 3 days before clinic visit. All participants randomized to the intervention arm also received a phone call on the day they missed a clinic visit and a home visit within a week of missed clinic visit.

For this study, two FGDs were conducted with all 15 *Mama Mshauri* 1 month after completion of the main study. Focus groups were conducted at the ICAP office and all *Mama Mshauri* received a transport stipend, and refreshments were provided during the sessions. Numbers were assigned to participants during the discussions and were used in quoting information during data analysis.

### Data collection

Fifteen *Mama Mshauri* participated in two focus group discussions held in October 2015 after completion of all study related activities. The first focus group included nine *Mama Mshauri* with unknown or negative HIV status (FG1), and the second focus group included six self-reported HIV-positive *Mama Mshauri* (FG2). Each FGD was facilitated by a female study staff member and a female note taker; the FGD with the HIV-positive* Mama Mshauri* lasted 3.5 h and the one with those of unknown status lasted 4.5 h.

The focus group staff used a semi-structured interview guide to facilitate discussion. Discussion topics included *Mama Mshauri’s* experiences with their clients throughout the intervention and with specific intervention procedures such as phone calls, SMS texting, home visits and clinic visits, and health education and adherence counseling. Additionally, participants were asked to discuss HIV status disclosure and behavioral-, social-, and structural-level issues surrounding HIV care and antiretroviral treatment during pregnancy. FGDs were conducted in Luo, audio-recorded, transcribed, and translated into English.

### Ethical considerations

The study protocol, data collection instruments, and consent forms were approved by the Columbia University Medical Center Institutional Review Board and the Kenya Medical Research Institute Ethics Review Committee. Written informed consent was obtained from all participants prior to study participation.

### Data analysis

Focus group data management and analysis were conducted with Atlas.TI (Version 1.0.38). Focus group transcripts were read repeatedly by the qualitative researchers. Two qualitative researchers independently reviewed focus group transcripts and developed a coding framework using applied thematic analysis, with codes developed and configured along lines of significant inquiry. Transcripts were coded by both researchers to identify relevant themes and categories. Codes were summarized, discussed, refined, and verified for consensus between the qualitative researchers.

## Results

Demographic characteristics of the fifteen *Mama Mshauri* are presented in Table 1. The median age of the participant’s was 37 years (IQR 31–43 years), 9 (60%) had secondary education, and 6 (40%) reported being HIV-positive. Qualitative data analysis showed generally positive experiences with the combination intervention, although participants also expressed concerns that arose during implementation. To assist in analysis, all intervention activities performed by the *Mama Mshauri* were categorized by the three major points of contact between *Mama Mshauri* and their patients, which included: 1) phone/SMS, 2) home visits, and 3) clinic visits. The study-specific flip chart was used across all major points of contact. Salient themes were identified from the experiences of *Mama Mshauri* across each part of the combination intervention, including: increased communication across various forms of patient contact and facilitated by the use of the flip chart, increased health education as a result of patient contact and flip charts, increased peer support across all forms of patient contact, client concerns about privacy and stigma across all intervention points, patient advocacy and assistance within a limited health system, and healthcare worker attitudes. Themes were then grouped into three broad categories: behavioral, social, and structural. Table 2 demonstrates the spread of qualitative themes across intervention points.

**Table 1:** Demographic characteristics of *Mama Mshauri* (*n* = 15)

Characteristic	Median (IQR) or Number (percent)
Age (years)	37 [31–43]
Ethnicity	
Luo	14 (93.3%)
Kisii	1 (6.7%)
Language	
Luo	15 (100%)
Education	
Secondary	9 (60%)
College	6 (40%)
Number of children ^*a*^	4 [2–5]
Place of delivery of last child	
Health facility	13 (86.7%)
Home	1 (6.7%)
Currently in a relationship	
Yes	11 (73.3%)
No	4 (26.7%)
Marital status ^*b*^	
Married, living together	5 (33.3%)
Not married, living together	1 (6.7%)
Married, not living together	2 (13.3%)
Not married, not living together	4 (26.7%) 2 single, 1 widow
HIV status (self-report)	
Positive	6 (40%)
Unknown/Negative	9 (60%)

^a^Number of children: 1 participant had no children

^b^Description of current relationship: 2 participants were single and 1 was a widow

**Table 2:** Qualitative themes across intervention points of contact

	Phone/SMS	Home Visit	Clinic Visit
BEHAVIORAL			
Increased communication and contact	√	√	√
Increased health education		√	√
SOCIAL			
Peer support	√	√	√
Privacy concerns	√	√	√
STRUCTURAL			
Patient advocacy and assistance			√
Healthcare worker attitudes			√

### Behavioral themes

#### Increased communication

SMS text and phone calls were used by *Mama Mshauri* to remind clients of their upcoming appointments. Mobile phone contact also allowed *Mama Mshauri* and clients to plan for any barriers to their appointment and discuss strategies and/or alternatives to ensure clients would make it to their upcoming appointments:

“Maybe the client was supposed to come on Wednesday but she is having some commitments that day, now you can even tell her to come on Tuesday, the day she is free so that she doesn’t miss [her appointment].” [FG2P4]

“Making phone calls as a reminder helped the women a lot. They would mostly say that I didn’t know where to leave my child but now that you have reminded me, let me go and tell the grandmother that I am having some journey tomorrow so that I leave him there with her […] It was always observed that these women were more punctual in the clinic compared to the ones who were not getting the reminders.” [FG2P3]

*Mama Mshauri* also recalled that the repeated SMS and phone reminders sometimes assisted newly-diagnosed clients in adjusting to their new HIV status:

“I would say that the SMS really helped many of my clients because you find someone is recently infected, she was recently diagnosed HIV positive, she is not used to appointments […] So when you keep reminding her, you make the clinic appointments easier for her to attend […] It made most of the clients accept their status very fast and attend the clinic visits.” [FG2P6]

Finally, the combination of interventions (SMS text and phone calls, home visits, and clinic visits) led to more frequent direct contact between patients and *Mama Mshauri*, which increased overall communication and facilitated other benefits such as increased health education, peer support, and patient advocacy, which are explored in more detail below.

#### Increased health education

*Mama Mshauri* conducted structured health educational sessions using PMTCT flip charts during home visits and clinic visits to deliver retention and adherence support and counseling. The study specific PMTCT flip charts were described as useful and comprehensive teaching aids, and covered a range of topics including HIV, mother-to-child transmission (MTCT) and PMTCT basics, ART counseling, HIV testing for family members, HIV status disclosure, serodiscordancy within couples, labor and delivery, postpartum and infant care, breastfeeding, and HIV testing and care for infants. The full list of topics covered in the flip charts can be found in Table 3. *Mama Mshauri* recalled that the combination of text and images made the charts easy to use and understand, and clearly communicated educational messages for mothers during both home and clinic visits:

“Okay, how the flip charts were of help to me, it was brief. When teaching a client, you will give education on something direct, after completing the topic, when you ask her, you will realize that she understood it very well.” [FG2P5]

“The flip chart we were using was really helpful because when you are teaching, she sees the pictures also. She sees what is going on. In the pictures she will even see the drugs; there is calendar, things like that.” [FG2P4]

**Table 3:** Flip Chart Topics

Flip Chart Topics	Session
HIV Basics	Throughout pregnancy
Mother to Child Transmission of HIV	Throughout pregnancy
PMTCT Basics	Throughout pregnancy
Staying Healthy During Your Pregnancy	Throughout pregnancy
Preparing to Start and Adhere to Lifelong ART	Throughout pregnancy
First Line Antiretroviral Regimen	Throughout pregnancy
Adhering to Your PMTCT Care	Throughout pregnancy
HIV Testing for Your Partner	Throughout pregnancy and postpartum care
HIV Testing for Your Children	Throughout pregnancy and postpartum care
Disclosing Your HIV-Status	Throughout pregnancy and postpartum care
Being Part of a Discordant Couple	Throughout pregnancy and postpartum care
Having a Safe Labor and Delivery	Term and early postpartum care
Taking Care of Yourself After Your Baby is Born	During term and early postpartum care
Continuing to Take Your ART While You Are Breastfeeding	During term and early postpartum care
Making Decisions About Future Childbearing and Family Planning	During term and early postpartum care
Feeding and Caring for Your New Baby	Postpartum care
Testing Your Baby or Child for HIV	Early infant follow-up visits
Caring for Your Child if He or She is HIV-Infected	If baby determined to be HIV-positive

Home visits also allowed more time for education and counseling, and the personal nature of home visits ensured that mothers would receive individualized attention from *Mama Mshauri* to address issues that had not come up during clinic visits:

“Some have problems talking to you in the facility or in the hospital but when you visit her at home, and then she is very open with you. She will be able to ask you questions, like ‘how do I breastfeed my child? How long do I breastfeed her? Do I give her porridge? And how do I take my drugs?’ All these are answered.” [FG1P3]

“Home visits are good because sometimes in the hospital, you don’t get enough time to teach someone, because she also has to go for her clinic, because she’s finished late, but at home you got the enough time to discuss.” [FG1P4]

### Social themes

#### Peer support

SMS text and phone reminders created a channel of communication between *Mama Mshauri* and clients, opening up spaces for other types of patient support and counseling:

“These SMS really assisted them. Sometimes she could also text me like ‘sister my drugs are over, what can I do?’ she’d send me such messages.” [FG1P6]

“At times if they are having a disagreement with the husband in the house, you could see they are sending you ‘please call me’ […] Then when I call you would hear her say, ‘surely sister, it was like this and this. Can you come and assist me?’” [FG2P1]

Home visits allowed *Mama Mshauri* insight into their clients’ lives beyond the walls of the clinic and the opportunity to provide additional support to family members. *Mama Mshauri* described home visits as valuable personal spaces to observe and provide support on issues such as medication adherence, nutrition, and the health of their other children. This provided the opportunity to provide holistic support to the entire family, an additional benefit not only to the mother and infant pair.

“When you go to visit a client at home, you will confirm whether the drugs she is supposed to give the child is being given to the child and how she is storing them, and also whether she taking her drugs.” [FG2P6]

“When we go to visit them, we are able to see other people. When she comes to the hospital we will only see her and the baby she is carrying. So I could go home at times and find that she is having other children as well. You find that when you are looking at other children, their health is not good. Some children at home looked malnourished. You will start educating her that she needs to go with those other children to the hospital at once to also know their status. Most of the time you find that one of the children that you suspected turns out to be HIV infected. If you had not gone there you would not have assisted that woman.” [FG2P2]

*Mama Mshauri* reported that meeting clients at their routinely scheduled clinic visits was helpful for clients because they received education and support from the *Mama Mshauri*, in addition to their already scheduled medical services. *Mama Mshauri* recalled that the privacy of the clinic space and their close relationships with clients resulted in clients feeling free to share their concerns or problems with *Mama Mshauri*.

“Clinics visits are of great help because… it would make you as *Mama Mshauri* be close with her more than the coordinator, because she can share with you even what the site coordinator is not aware of because you are her close friend.” [FG2P1]

“Maybe they would come to clinic with a problem which could not be solved at home, and maybe something which she could not open up and share with you at home that can interfere with her CD4 and at [in the clinic] she might open up and share… Clinic helps us and clients also get relieved. When she is free she can open up and share with you something which was disturbing her mind which can also interfere with her adherence.” [FG2P6]

While all *Mama Mshauri* characterized their relationships with clients as supportive, several HIV-positive *Mama Mshauri* explained how their HIV status positioned them to better understand their clients, due to their similar life experiences. Additionally, several HIV-positive *Mama Mshauri* described strengthened relationships with clients upon disclosing their own HIV status:

“I can say that it is helpful if *Mama Mshauri* is someone with HIV because first and foremost, you have your own experience, when you tell a client that a child is normally breastfed for six months without introducing any other food, you also have your own experience because you also did that, you can tell her many things through your experience, when you tell her how to swallow drugs, you also have the experience.” [FG2P6]

“When I disclosed my status to my client, she was shocked and also encouraged; I told her, ‘do you know what is happening? I don’t want you to go through what I went through, I went through that because I didn’t know and I don’t want it to happen to you.’ She was shocked and later became very strong, she said that these sisters are also like that, she became strong and I also encouraged her, she now comes to the clinic without us reminding her.” [FG1P1]

While similar life experiences helped HIV-positive *Mama Mshauri* to relate to their clients, most *Mama Mshauri* described other factors as more important to their clients’ well-being, such as their own knowledge and dedication to their jobs:

“You can be positive and the client is also positive [but if] your heart is not dedicated, you have not helped her. You can be negative and the client positive and very helpful… Therefore I can suggest that being *Mama Mshauri* doesn’t require the same status with the client.” [FG1P2]

“Above all, knowledge is everything. If she is trained with relevant skills and information then there a way in which she can train the positive to live a good life. I think knowledge is better than just the HIV status.” [FG1P7]

#### Concerns about privacy and stigma

Concerns about privacy and stigma were frequently mentioned across all intervention methods, including SMS text and phone calls, home and clinic visits, and the use of the flip chart. *Mama Mshauri* relayed their strategies for navigating client concerns regarding privacy and confidentiality at various levels of the intervention.

##### Mobile phone intervention

SMS text and phone call reminders elicited privacy concerns from clients who had not yet disclosed their status to their partners. Because women and their partners often share one phone, *Mama Mshauri* recalled many varied strategies they had developed over the course of the intervention to ensure that they would be able to contact their clients. These strategies were often planned in advance between *Mama Mshauri* and their clients to allay any concerns regarding privacy or exposure. For SMS reminders, the primary strategy *Mama Mshauri* employed was to formulate generic SMS reminders that omitted any specific references to PMTCT.

“On this SMS we sent them, it was a general message. It was a message that if one could read then he could not be able to understand why it was sent such a message.” [FG1P9]

“We were never specifying, we would say, ‘I would remind you to come to clinic on such and such a day but we were never specifying CCC clinic [HIV Chronic Care clinic] or MCH.’ So it was her that knew the exact things because we had talked to her and she knew it in her own mind.” [FG1P2]

Other SMS-related strategies included clients assigning the *Mama Mshauri* false names in their mobile phones, so that only the client would know what their message signified. Other *Mama Mshauri* constructed stories for their clients in preparation for their husbands’ inquiries:

“I used to advise them… go and tell him that lately there is an arrangement in the clinic, people are now being reminded to go back to clinic on their appointment dates… that it is a new organization that is doing this. Every woman who is coming to the clinic is reminded to take back the child to the clinic at appropriate time, if it is a pregnant woman, for her to come at the appropriate time as appointed. That is the way I was helping that situation.” [FG2P3]

However *Mama Mshauri* reported that for some clients, the risk of exposure was too great, and contacting them via SMS was simply not possible:

“When she has not disclosed her status to her husband, she will not agree because they are also sharing the phone. It can bring a lot of problems because if you send her the SMS… He will start investigating why she is receiving the SMS or he might even start asking other women if they are also receiving SMS. If he realizes that his wife is positive, he will start bringing a lot of trouble. We had other women who did not accept to receive SMS.” [FG2P6]

*Mama Mshauri* also developed strategies with their clients to facilitate phone call reminders and support, and reported that some clients preferred to receive only phone call reminders (and not receive SMS text reminders) to maintain privacy. *Mama Mshauri* and their clients would agree upon the particulars of phone calls, such as what times were best to call and which phone was best to use.

“The time for making calls is an agreement between you and the client. She is the one who tells you that you can even call me at night, call me even in the morning, or she can set you free and tell you that she is free the phone is hers; call me whenever you feel like. So it is an agreement between you and the client.” [FG2P6]

While *Mama Mshauri* reported their strategies for maintaining SMS text and phone call reminders were largely well received and useful, some *Mama Mshauri* relayed stories of potential family conflict arising from SMS and phone reminders.

“If you send her a message, or make a phone call the husband was the one with the phone; receiving all the communication. You call and say greetings father of the baby because we knew that the husband is the one who had the phone, I would just like you to remind madam to come to the clinic tomorrow. But when the husband arrived at home a fight would start between him and the wife.” [FG2P1]

##### Home visits

As with the SMS text and phone reminders, the most significant challenge for home visits was privacy. *Mama Mshauri* described client concerns related to privacy, disclosure, and fear of being stigmatized by the community. Home visits to women who had not disclosed their status to their husbands and/or families were simply not feasible:

“Okay, the challenge we faced in home visits concerning the people who had not disclosed to their husbands. If you have not disclosed there was no way we could come to visit you. So we would just finish that in the clinic. It was not easy. So we just had to finish that in the clinic.” [FG2P5]

“She requested that we don’t go and visit her until she moves to her own home because the neighbors may see her and ask her what that woman came to do because at the hospital if they see us talking to any person they just start asking. They fear us.” [FG2P3]

For some *Mama Mshauri*, visiting clients who had not disclosed resulted in potentially tenuous situations for both clients and *Mama Mshauri*:

“During my home visits we would go to their homestead, and I find her with many people, I could not talk to her or even share with her.” [FG1P1]

“They accepted to be visited but when you get there you just whisper. Those slum houses are funny; anyone talking on the other side can be heard on this other side.” [FG1P9]

To address concerns of privacy in home visits, *Mama Mshauri* described the various strategies they employed, including setting very specific times to visit, fabricating relationships with the client, and meeting outside the home.

“I had a client who said that I was her sister. From the first day she was enrolled she went and told her husband that she had met her long time sister in the hospital working there. It was her husband who was the first one to call me, ‘hello I am so and so the husband to your sister’. So that is how I kept the confidentiality.” [FG1P7]

“So there was this bush around her homestead and we went there and had a good discussion but unfortunately we were not aware that it was coming to rain. So when it started to rain, we were rained on and I remember asking her ‘what will we now do?’ and she said ‘no, do not come back to my home, we can be meeting here from now’. So that is another challenge I saw as we do these home visits.” [FG1P6]

Some *Mama Mshauri* recalled how their strategies to maintain privacy often necessitated the omission of conspicuous components of the home visit, such as the flip chart.

“I did not use [the flip chart] on many occasions during home visits. At times you can go and find her with someone, may be many people go to that house and the moment someone sees you with a flip chart on the table, the person will just know that there is something, there is something going on, so you can go with it for home visits and fail to use it.” [FG2P2]

Additionally, in spite of their best strategies, *Mama Mshauri* noted that just their presence in the village or home would prompt questions from family or community members:

“People know so much about HIV that when they see us asking someone’s house they can even ask you ‘she hasn’t taken her medication?’ that is what they will ask you. ‘She has defaulted? She was taking her drugs well, why has she disappeared?” [FG1P9]

##### Clinic visits

*Mama Mshauri* noted that patient concerns about stigma or being exposed as HIV-positive at the clinic were frequently mentioned by clients and that these fears were sometimes reinforced by a lack of privacy at the clinic, sometimes due to indiscreet healthcare workers, and sometimes due to the physical set-up of the clinic rooms and procedures:

“There are some clinicians when they see a client, he/she might ask that are you still breastfeeding your baby? ‘You want to give the baby your HIV?’ meanwhile that is a corridor and people are seated there, so you see people standing to look at the client, they want to see the person who wants to infect her baby with HIV.” [FG2P1]

“I can say that there is little confidentiality because of the building structure in the facility… Rooms are labeled and everybody knows the name of the room you have entered, these days people are also educated and clever, you will also find that we have been given clothes with writings on the back, so each time you are entering the room and coming out, they are learning something about your movement.” [FG2P2]

##### Flip charts

As mentioned previously, *Mama Mshauri* reported that the flip chart was potentially ill suited for the environment of home visits, since the clear pictorial representation of the subject matter could potentially enable bystanders to learn the clients’ HIV status by simply seeing the images.

“The way [the flip chart] was designed made it easy to the user. But on the other hand, it was difficult to the user because of those pictures like the part of taking drugs; there is a picture of someone with a cup when swallowing a drug. When teaching someone on how she should be taking drugs and someone [might come in] and [see] this picture about how to take the drugs… she will be having some fear that whoever is seeing this will realize that she is on drugs.” [FG1P1]

However flip charts were utilized and well-received consistently in clinic visits, where clients and *Mama Mshauri* were assured more privacy:

“I did not use [the flip chart] on many occasions during home visits… But we never failed to use it on clinic visits because were in conducive environment and there was privacy.” [FG2P2]

“[Flip charts were used with] high frequency especially in the clinic visits. Where there is freedom. Where the clients have the freedom and even if you use a flipchart, she doesn’t have any problem.” [FG1P7]

### Structural themes

#### Patient assistance in a limited healthcare facility

When accompanying clients at their routinely scheduled clinic appointments, *Mama Mshauri* were positioned to act as patient advocates, assisting their clients to navigate efficiently through their medical appointments:

“We were helping with facility navigation at all times and linking them with antenatal or postnatal services such as family planning. You would tell her that now you should be doing your family planning and you link her to the nurse and tell her to wait for you. It is not that you will skip the line for her, but you just have to make sure that she gets the service. Even if she goes to the laboratory, you can sometimes escort her to the laboratory and talk to the laboratory technologist to give her priority and attended to her then she comes back for the remaining services.” [FG2P5]

*Mama Mshauri* often had to contend with the limitations of the healthcare system, such as a lack of space for visits and understaffing:

“In this facility we did not have an office space…So we would borrow a space within the comprehensive care clinic (CCC) or MCH clinic while the clinician was in the other end. But at times the room was occupied you had to wait. So we experienced a lot of challenges. Personally as *Mama Mshauri* sometimes I was forced to go and talk to my client under a tree that was not good thing. Sometimes ants would bite you, and people are also looking at you this really interfered with the clinic visit session.” [FG1P2]

“The other challenge was that facility providers were absent. The providers were different sometimes you bring a client for PCR testing you find the nurse is not there. You will be forced to go and look for someone else to sweet talk him or her in order to get the PCR sample taken.” [FG2P6]

*Mama Mshauri* noted that staff shortages, limited resources, and mismanaged paperwork often lead to long wait times and delays in care:

“There are no test kits, she ends up going back home without that service, she will make several visits but still there are no test kits and when she comes when there are test kits there will be no one who is ready to offer her the service… the client will go back home without the service. This normally happens.” [FG2P2]

“What can prevent women and mothers also from accessing health services in the hospital and frustrate their efforts of coming is that a woman comes in the morning and will leave at 2 pm because her file went missing [...] Next time she comes back very early in the morning again and then her file is still missing, it cannot be found […] she will become frustrated and decide not to take drugs anymore because of the file, she doesn’t go home with her file, her file remains in the hospital, now where did the file go?” [FG2P4]

“This woman was coming to clinic when pregnant. There were no test kits and now she was not tested early so that [if she tested positive] she could start taking drugs to prevent the fetus from getting infected. By the time they will be doing that, may be they will find when the fetus is already infected. When there are no re-agents for CD4 test, the mother might be due for ART initiation and she cannot be helped, they will end up telling her to come back later of which when she comes still she will be put on another drug which cannot help her because of lack of supplies.” [FG2P6]

Additionally, *Mama Mshauri* reported that while they tried to mediate clients’ time constraints and potentially long waiting times at the clinic, they felt that clients expected a great deal from them, and *Mama Mshauri* were not always able to meet these high expectations.

“You would experience a challenge because clients having known that you are an insider… She will be on your neck to help her jump the line, sometimes she could be in 6 weeks and in that stage she will be required to visit like five rooms. She expects you to help her in all those stages and those on the line are also making noises. So it is a challenge, because they know very well you are well known in the facility, so she turns up at eleven and you know the CCC ends at noon.”[FG1P4]

#### Health care worker attitudes

*Mama Mshauri* cited healthcare worker attitudes and behavior as a potential barrier to women’s engagement in the PMTCT cascade, describing peer educators and clinicians as sometimes hostile and insensitive to their clients, shaming patients for their behaviors. This is important given their role in service provision and provision of psychosocial support for pregnant and postpartum women.

“You might find if a peer educator harassing a client on the bench ‘look at how you want to kill the baby, you had just killed the other one and here you are killing this as well? Look at this mother, she killed another child and here she is killing another one? This one shouldn’t be assisted.’ That day I saw that client stood up crying and she left.” [FG1P7]

Having an insider perspective to health care worker attitudes towards clients, the *Mama Mshauri* would discuss some of their observations with the study coordinator who was in a position to address them with the clinic-in-charge. This was helpful because it enable *Mama Mshauri* to act as a bridge between clients and health care workers, and also to let the HCW understand the effect of their actions on the clients.

“Their attitude is not good and even how they deal with clients is not good, sometimes the clinicians working in PMTCT work closely with peer educators, when a peer educator has a grudge with the client, he/she will go and say nasty things about the client to the clinician [such as] handling that client is very difficult, that client like missing appointments… The clinician is going to treat the client badly because of the nasty things he/she has heard from the peer educator.” [FG2P6]

“Yes people can get busy and then you arrive a little bit late and then you humbly tell the peer educator there that I arrived late, maybe he/she had stopped taking files, you will ask her/him to help you get your medicines, he/she can chase that client away and say that now that you are late, just go back and come back the following day and maybe the client will not have that opportunity of coming back tomorrow because she will be much busy than she was today. Where we work, people working in the hospital can make clients stop getting the services that they should be getting.” [FG2P2]

## Discussion

This is the first study to evaluate the experiences of lay health workers administering a combination intervention to improve retention among HIV-positive women initiating PMTCT services and their infants in Kenya. Study findings show lay health workers played a critical role supporting mothers in PMTCT services with this combination intervention, across a range of behavioral, social, and structural factors that have been evidenced to influence the uptake and retention of PMTCT services, including improved communication and contact, health education, peer support, and patient advocacy and assistance. Findings also identified barriers to the uptake and implementation of the intervention, as well as barriers to general uptake and retention in PMTCT services, such as concerns about privacy and stigma, and the limitations of the healthcare system including healthcare worker attitudes. Overall, study findings indicate that *Mama Mshauri* found the interventions to be feasible, acceptable, and well-received by clients. However, concerns about privacy need to be carefully considered when implementing on a wide scale.

Study findings demonstrate the ability of *Mama Mshauri* to address barriers to PMTCT care across behavioral, social, and structural levels. Phone and text reminders increased communication between clients and *Mama Mshauri* and enabled clients to prepare to attend their upcoming appointments. These findings align with previous research on mobile phone interventions, which has shown increased communication as a result of mobile phone reminders and two-way mobile phone communication can increase postnatal clinic attendance and infant follow-up visits among HIV-positive mothers [16, 38, 39]. Additionally, *Mama Mshauri* in this study had multiple points of contact with their clients, including phone calls and text messages, and home and clinic visits, which lead to increased opportunities for health education and peer support. For example, *Mama Mshauri* were able to deliver MCH and ART education to mothers across these contact points and able to support clients in unique ways, such as assisting women to find childcare in order to make her appointment, or noticing malnourishment among her other children at a home visit. Previous research has demonstrated that peer support is associated with improved PMTCT retention [20, 40], and that home visits provide a unique opportunity for lay workers to offer tailored support and education to patients [41], and mobile phone interventions have also indicated that peer support is an important component of communication between health workers and clients [38]. In this study, some *Mama Mshauri* attributed their close relationships with their clients to their own HIV status disclosure and the mutual recognition of shared experiences. This is an important finding, which is supported by previous research showing how mutual shared experiences between peer counselors and clients can decrease levels of depression and feelings of isolation among new mothers, improve infant well-being and partner relationships, and facilitate adherence to ART [42, 43]. Findings also show how *Mama Mshauri* were able to assist clients during their clinic visits by acting as patient advocates and performing a variety of duties, such as liaising with clinic staff, using the flip chart for health education, or providing peer support.

At the same time, findings revealed the significant challenges *Mama Mshauri* encountered at multiple points in the intervention, particularly regarding privacy and stigma. Our study provides important information from the perspective of lay health workers regarding the strategies used to address privacy concerns, including when to send text messages, make phone calls, and schedule home visits, being ready with an alternate explanation for the health workers’ presence in their clients’ homes, meeting outside the home or outside the clinic, and amending the protocol for health education, such as omitting the use of the flip chart during home visits. While these strategies underscore the ability of lay health workers to adapt the intervention to their clients’ needs, such findings highlight a need for future interventions to consider the privacy implications of such a comprehensive intervention. Fear of stigma and privacy concerns have been established as major barriers to the uptake and retention of PMTCT services [8, 44–46], and other interventions have noted the complexities of maintaining privacy while communicating with clients, including mobile phone interventions and home visits [16, 47, 48]. Future interventions must have plans to address privacy concerns ahead of time, including alternate plans for contacting and meeting clients, and extensive training and mentoring of lay health workers to protect patient privacy in the field. Additionally, some research has shown community health interventions can decrease stigma through HIV education and counseling, including providing the community with positive perceptions of people living with HIV [49].

*Mama Mshauri* also discussed significant challenges related to the limitations of the health system. Reports of supply and staff shortages, lack of space, and mismanaged paperwork highlighted the important role *Mama Mshauri* played as patient advocates, however *Mama Mshauri* were not always able to circumvent institutional barriers to care. Findings on healthcare worker attitudes further indicate that healthcare workers may require additional support and mentoring. Previous research has suggested that healthcare workers working in HIV care face especially high rates of stress and burnout [46, 50]. Many previous studies have described the limitations of the health system in resource-limited settings, and the potential barriers to care created by staff shortages, lack of supplies and medication [9, 44, 46], as well as the ways in which lay health workers can alleviate the burden to patients by reducing wait times, assisting with patient navigation, addressing patient issues at home and clinic visits, and acting as an intermediary between patients and the healthcare providers [32, 41, 51–53]. The *Mama Mshauri* also highlighted negative attitudes of healthcare workers to clients. This is important since the health care workers role is not only to provide healthcare services but also to provide psychosocial support. This underscores the importance of training and mentorship on patient centered care to ensure that health care workers are supportive and respectful to clients [54].

Several study limitations should be noted. By design, qualitative studies aim to gain in-depth insight and diversity of opinions from small samples, limiting generalizability of findings. This study intended to collect insight into the experiences of lay health workers delivering a comprehensive intervention designed to improve retention among HIV-positive mothers in PMTCT services. Focus group dynamics may have led to the overrepresentation of views of certain participants and underrepresentation of others. This study also has several strengths. This is one of the first studies to evaluate the experiences of lay health workers administering a combination PMTCT intervention, and all *Mama Mshauri* who delivered the intervention were included in this study.

## Conclusions

This study assessed the experiences of lay health workers administering a combination of evidence-based interventions to improve retention among HIV-positive women initiating PMTCT services and their infants in Kenya. Findings demonstrate the fundamental role lay health workers play in supporting mothers engaged in PMTCT services by addressing behavioral, social, and structural factors associated with retention in care. Study findings also highlight the need for future interventions to include strategies to ensure privacy and decrease stigma within communities and facilities. This study adds important insight to the small but growing body of research on lay health worker experiences in HIV and PMTCT care.

## Abbreviations

ART: Antiretroviral therapy
CCC: Comprehensive care clinic
FGD: Focus group discussion
LMIC: Low and middle-income countries
MCH: Mother and child health
MIR4Health: The Mother and Infant Retention for Health Study
MTCT: Mother-to-child transmission
PMTCT: Prevention of mother-to-child transmission
SMS: Short message service (texting)
